# PK-PD Analysis of Marbofloxacin against *Streptococcus suis* in Pigs

**DOI:** 10.3389/fphar.2017.00856

**Published:** 2017-11-20

**Authors:** Zhixin Lei, Qianying Liu, Bing Yang, Haseeb Khaliq, Jiyue Cao, Qigai He

**Affiliations:** ^1^Department of Veterinary Pharmacology, College of Veterinary Medicine, Huazhong Agricultural University, Wuhan, China; ^2^National Reference Laboratory of Veterinary Drug Residues and MAO Key Laboratory for Detection of Veterinary Drug Residues, Huazhong Agricultural University, Wuhan, China; ^3^State Key Laboratory of Agriculture Microbiology, College of Veterinary Medicine, Huazhong Agricultural University, Wuhan, China

**Keywords:** marbofloxacin, *Streptococcus suis*, pharmacodynamic, pharmacokinetic, optimal dosage, fluoroquinolone

## Abstract

Marbofloxacin is a fluoroquinolone antibiotic and highly effective treatment for respiratory diseases. Here we aimed to evaluate the *ex vivo* activity of marbofloxacin against *Streptococcus suis* in pig serum, as well as the optimal dosages scheme for avoiding the fluoroquinolone resistance development. A single dose of 8 mg/kg body weight (bw) was administrated orally to healthy pigs and serum samples were collected during the next 72 h. Serum marbofloxacin content was determined by high-performance liquid chromatography. We estimated the C_max_ (6.28 μg/ml), AUC_0-24 h_ (60.30 μg.h/ml), AUC_0-∞_ (88.94 μg.h/ml), T_1/2ke,_ (12.48 h), T_max_ (0.75 h) and Cl_b_ (0.104 L/h) of marbofloxacin in pigs, as well as the bioavailability of marbofloxacin (94.21%) after a single 8 mg/kg oral administration. We also determined the pharmacodynamic of marbofloxacin against 134 *Streptococcus suis* strains isolated from Chinese cities in TSB and serum. These isolated strains had a MIC_90_ of 1 μg/ml. HB2, a virulent, serotype 2 isolate of *SS*, was selected for having antibacterial activity in TSB and serum to marbofloxacin. We determined the minimum inhibitory concentration (MIC, 1 μg/ml in TSB, 2 μg/ml in serum), minimum bactericidal concentration (MBC, 4 μg/ml in TSB, 4 μg/ml in serum), and mutant prevention concentration (2.56 μg/ml in TSB) for marbofloxacin against *Streptococcus suis* (HB2). In serum, by inhibitory sigmoid E_max_ modeling, the AUC_0-24h_/MIC values for marbofloxacin against HB2 were 25.23 (bacteriostatic), 35.64 (bactericidal), and 39.71 (elimination) h. Based on Monte Carlo simulations, the predicted optimal oral doses of marbofloxacin curing *Streptococcus suis* were 5.88 (bacteriostatic), 8.34 (bactericidal), and 9.36 (elimination) mg/kg.bw for a 50% target attainment ratio, and 8.16 (bacteriostatic), 11.31 (bactericidal), and 12.35 (elimination) mg/kg.bw for a 90% target attainment ratio. The data presented here provides optimized dosage information for clinical use; however, these predicted dosages should also be validated in clinical practice.

## Introduction

*Streptococcus suis* (*SS*) is an important swine industry pathogen that could cause significant economic losses worldwide. *SS* is also considered an emerging zoonotic pathogen with the potential to cause disease (e.g., meningitis, septicemia, pneumonia, arthritis, and endocarditis) in humans and pigs (Feng et al., 2014; Goyette-Desjardins et al., 2014). Based on capsular antigens, there are 35 *SS* serotypes, among which serotype 2 is the most virulent, as well as being the dominant pathogenic serotype (Gottschalk et al., 2010a, 2013; Feng et al., 2014). Large outbreaks of *SS* serotype 2 have occurred in China in 1998 and 2005, causing high morbidity and mortality in pigs and humans (Yu et al., 2006; Song et al., 2011). Because *SS* vaccines are either unavailable or unsuitable, antimicrobial agents are used to treating *SS*. *SS* resistance to antimicrobial agents (macrolides, lincosamides, sulfonamides, and fluoroquinolones) has been reported (Varela et al., 2013; Yang et al., 2015). Therefore, it is urgent need to set up an optimal dosage scheme for *SS* treatment.

A third-generation synthetic antibiotic fluoroquinolone, marbofloxacin (MBF), has a wide range of bactericidal effect for Gram-negative, partial Gram-positive pathogens and Mycoplasma (Haritova et al., 2006b; Grandemange et al., 2012; Vallé et al., 2012; Tohamy and El-Gendy, 2013; Lei et al., 2017a). MBF is recommended to orally or parenterally administrated for pigs and bovine diseases in the respiratory and digestive tract (EMEA, 1996; Ding et al., 2010). It has shown strong bactericidal activity against pathogenic isolated from respiratory tract infection, urinary tract infection and alimentary tract in animals such as pigs and pets (Yohannes et al., 2015; Serrano-Rodríguez et al., 2017). In addition, among the fluoroquinolones, MBF is unusual because it’s safe, concentration-dependent mode of action allows the use of a single, short-term, and high-dose that provides both good efficacy and reduction in the development of target pathogenic resistance (Giguère et al., 2004; Dowling, 2006; Ahmad et al., 2015, 2016).

It has been studied for the pharmacokinetics (PK) research of MBF in different kinds of animals, including dogs, pigs and so on, showing high concentration, rapid and extensive distribution in the peripheral compartment or tissue and a high bioavailability closed at 100% after extravenous administrations (Waxman et al., 2001; Schneider et al., 2004; Albarellos et al., 2005; Ding et al., 2010; Sidhu et al., 2010; Lei et al., 2017a). These PK profiles make the MBF an attractive option for treating the herd of food-producing animals and effective in treating serious infections (e.g., septicemia and gastroenteritis) caused by Gram-negative or some Gram-positive aerobic bacterium (Garch et al., 2017). MBF has been shown to have excellent PK characteristics after extravenous administration, with higher concentrations in tissues, low serum protein binding (<10%) and having a long half-life with widespread distribution throughout the body (Haritova et al., 2006a,b; Jian et al., 2015). PK and pharmacodynamics (PD) properties can be used to predict the effectiveness of antibiotics, suggesting an appropriate dose that maximizes the effectiveness of therapy while minimizing the development of bacterial resistance (Drusano, 2004). However, there is limited PK and PD data addressing MBF against *SS* serotype 2.

This could be a powerful tool to link dosage scheme to clinical effects using PK-PD modeling (Diloreto, 2014). Moreover, it is necessary to adjust the dosing regiments for clinical treatment while reducing the occurrence of resistance to antimicrobial drugs (Burgess, 1999). PK-PD modeling approaches were used to establish a dosage schedule best suitable for promoting the eradication of bacteria and reveal the relationship and quantify the efficacy of the antibiotics against the bacterium in target animals. As a result, it could provide optimal dosage schemes and decrease the hazard of resistance progress and determined carrier status with PK-PD modeling (Drusano, 2007; Nielsen and Friberg, 2013). As an effective tool to assess the optimal dosage scheme and prevent resistance progress, PK-PD modeling has been performed in the new antimicrobial compounds development procedure by the European Medicines Agency (EMA) and Food and Drug Administration (FDA) (Lei et al., 2017a; Yan et al., 2017).

The antibiotic concentrations investigated in the target site of animals were more and more prevalent. For *Escherichia coli* infection in pigs, MBF was studied in ileum content, which demonstrated that the concentration in ileum was much higher and more authentic than in serum in previously published reports (Wang et al., 2016; Lei et al., 2017a; Yan et al., 2017). The pulmonary epithelial lining fluid (PELF) of the lungs is widely regarded as the target site of *SS* infection in mammals. Similar to *Pasteurella multocida, SS* is a strictly extracellular pathogen (Rodríguez-Ortega et al., 2008; Mandanici et al., 2010; Haas and Grenier, 2015) and mainly localizes to the PELF. Although drug concentrations in the PELF greatly exceed those in the serum, the high PELF drug concentrations might be caused by cell lysis during the bronchoalveolar lavage procedure to collect PELF (Kiem and Schentag, 2008; Kiem and Schentag, 2014). Moreover, the difficulty of measuring PELF concentration could also be the most important reason for a proper PK driver selection and the technique might be labor intensive and not applicable for measuring PELF concentration (Nielsen and Friberg, 2013). Thus the measured concentration from site of plasma might be a suitable final target tissue for PK-PD analysis (Toutain et al., 2015, 2016). Furthermore, the drug concentrations in the serum are suggested to use to calculate reliable dosage regimens.

To our knowledge, no previous study has integrated the data of PK and *ex vivo* PD for MBF against *SS* in pigs. Although the EMA recommends a dosage regimen of 2 mg/kg for 3–5 days, as a single dosage of 8 mg/kg has been shown to be effective for the treatment of respiratory diseases (e.g., *Actinobacillus pleuropneumoniae*) (Schneider et al., 2014; Grandemange et al., 2017). Therefore, in this study, we administered a single 8 mg/kg oral dose of MBF and the rational dosage was established and verified by using PK-PD integration modeling.

## Materials and Methods

### Chemicals

Marbofloxacin standard with a purity over 98% was received from Dr. Ehrenstorfer GmbH (Augsburg, Germany). MBF powder a purity over 97% (No. 201104006) was provided by Wuhan Huishen Biotechnology, Co., Ltd. The MBF solution (40 mg/ml) was prepared with normal saline added 0.1% acetum solution. The chemical agents were all high-performance liquid chromatography level and the other solvents were also analytical level in this study. Each isolate strain was subcultured for over three times to reach stable growth in tryptone soya broth (TSB) and tryptone soya agar (TSA; Qingdao Hai Bo biological Technology, Co., Ltd.) with 5% newborn calf serum (Zhejiang Tianhang Biotechnology Co., Ltd.).

### Animals and Treatment

A number of 8 healthy pigs with four females and four males were selected from 15 to 20 kg and from 8 to 10 weeks for this study and provided by the Hubei Provincial Laboratory of the Public Center for Animal Services. The animals received feed and water without antibiotics *ad libitum* in the separation fence during the experimental period, and the premixes were devoid of polyvalent cations affected by MBF uptake, such as aluminum, magnesium, calcium, iron, etc. In addition, pigs were fasted overnight and kept for over seven days to acclimate before the formal experiment study. This study was approved and permitted by the Ethical Committee of the Faculty of Veterinary Medicine from Huazhong Agricultural University. In addition, all of the animal care and experimental protocols from this study were performed in accordance with the Guide to the Care and Use of Laboratory Animals of Hubei Provincial Laboratory Animal Public Service Centre with a permit number SYXK 2013-0044.

### MIC Determination for Isolated *SS*

One-hundred-thirty-four *SS* strains were isolated from Chinese pigs (from Henan, Sichuan, Hubei, Anhui, Hunan, Jiangxi, and so on) from 2016 to 2017. From these, we selected the *SS* HB2 strain (serotype 2) to detect the susceptibility effect of MBF *in vitro*. The *SS* HB2 strain was selected because its minimal inhibitory concentration (MIC) to MBF was similar to the MIC_90_ of MBF against *SS* (134). These strains were authenticated with polymerase chain reaction (PCR). Before MIC detection, these isolates were subcultured at least three times in TSB and TSA. All isolates were kept at -80°C (Lei et al., 2017b).

Susceptibility detection (MIC) for MBF against *SS* was conducted with the agar dilution method which was based on the guidance of CLSI (Jorgensen, 1993; Lei et al., 2017c). The strains (2–4 μl) with 10^8^ CFU/ml were inoculated into TSA including 5% newborn calf serum, with two-fold serial dilutions of marbofloxacin (0.125–64 μg/ml). Those isolates with MIC values over 64 μg/ml were re-tested using a broader range of MBF dilutions. Inoculated plates were kept at 37°C for 48 h. The MIC value was considered the lowest drug concentrations that caused complete growth inhibition by the naked eye. *E. coli* (ATCC 25922) was performed as a quality control (QC) strain to check the results of the above susceptibility testing.

### Detection of MPC, MBC and MIC in TSB and Serum

For MIC and minimal bactericidal concentration (MBC) determination of HB2 *in vitro* and *ex vivo*, it was performed using the microdilution technique following the guidelines of the CLSI, and MBC was tested by using inoculating the supplemented TSA with 100 μl of suspension received from the initial MIC detection with no distinct bacteria. The MBC value was considered the value at the lowest concentration of MBF with inhibiting bacterial density by 99.9%.

The mutant prevention concentration (MPC) of MBF was tested using the agar dilution method. The10^10^ CFU/ml of *SS* (HB2) was prepared to test the MPC on TSA plates (Blondeau et al., 2010). Then, the suspension of *SS* was unfolded onto TSA, containing serial dilutions of MBF (1–32 MIC); the MPC was the value at the lowest concentration with inhibiting bacterial growth at 37°C for 96 h.

### Growth and Killing-Time Curves *in Vitro* and *ex Vivo*

The isolate of HB2 was selected to test the growth-time curve of *SS* by optical density (OD_600 nm_). The growth curves of HB2 in serum and TSB were performed at 0, 2, 4, 6, 8, 10, 12, and 24 h using optical density.

According to the MIC value (1 μg/ml) of HB2 to MBF, TSA plates containing various MBF concentrations in the range of 1/4 to 32 MIC were prepared. For the vitro killing-time curve, 10^6^CFU/ml bacteria-containing suspension fluid was diluted to obtain with normal sterile saline. Then 100 μl dilute samples were spread onto the TSA plates at different time point including 0, 2, 4, 6, 8, 10, 12, and 24 h. Finally, these samples were cultivated at 37°C for over 48 h.

For the killing-time curves in *ex vivo* (MBF in serum received at various time points from 0 to 24 h in PK study), the bacteria (10^6^ CFU/ml) were cultivated with serum samples containing various MBF concentrations. The *ex vivo* killing-time curve was administrated to accommodate an appropriate PD model using the hypothesis that a logarithmic decrease of bacteria amounts under MBF concentration according to the incubation time with the inhibitory sigmoid E_max_ model.

### Animal Study

Eight pigs with four male and four female weighing 16–20 kg and aged 5–6 weeks were selected in this study. Each pig was administrated MBF (8 mg/kg) by oral administration. After a radical washout period (2 weeks), the pigs received MBF by intravenous injection (i.v, 8 mg/kg). The blood samples (5 ml) were collected at 0, 0.25, 0.5, 1, 2, 4, 6, 8, 10, 12, 24, 36, 48, 72 h after i.v and oral administrations.

### Blood Sample Extraction

The samples were collected with an anticoagulant and then centrifuged for 10 min at 3000 rpm to acquire the serum. 2 ml of dichloromethane was added to 0.5 ml of serum, the tubes vortexed for 2 min, and centrifuged for 10 min at 5000 rpm. The above procedures were repeated to perform twice. The dichloromethane fluid was taken to a clean tube and evaporated with nitrogen in a thermostat water bath at 60°C. A portion of the mobile phase (0.5 ml) was used to dissolve the dried sample. The dissolved samples were filtered through the membrane filters (0.22 μm) and analyzed by HPLC with UV for detection.

### MBF Binding to Serum Protein

Serum protein binding of MBF was determined in triplicate on each of nine pooled blood samples, harvested at predetermined times from the eight pigs used in the PK study. The samples were centrifuged at 3000 rpm for 10 min to obtain the serum. For each sample (serum), the total concentration of MBF was determined as follows. Samples were centrifuged for 10 min at 4000 *g* using an Amicon Ultra Centrifugal Filter (Ultracel 10kD; Millipore Limited, Watford, Hertfordshire, United Kingdom) and MBF concentration re-determined on the ultrafiltrate.

### HPLC Methods Validation and PK Analysis

The extraction samples in serum were analyzed using a C18 reverse-phase column (250 × 4.6 mm, i.d., 5 μm, Agilent, United States) and HPLC, which was performed with a detection wavelength of 299 nm at 30°C for the column. The mobile phase including 0.1% formic acid (phase A) and acetonitrile (phase B) (v:v, 18:82) with a mobile phase flow rate of 1 ml/min. In addition, the injection volume was 20 μl. The HPLC method validations of MBF in serum were analyzed by the standard external method. The linear range for the standard curve of MBF ranged from 0.05 to 10 μg/ml in serum was detected by HPLC, and the linear regression, curve recovery and coefficient of variation were calculated. The recovery ratios were the specific values of calculated peak area in serum to standard with different drug concentrations (0.05, 0.1, 0.5, 1, 5, 10 μg/ml). The LLOD was the lower detected concentration at the value of the signal to noise ratio (S/N) > 3 in serum. The LLOQ was the lower detected concentration at the value of S/N > 10 in serum.

Pharmacokinetics parameters were calculated from serum MBF concentrations with WinNonlin software (version 5.2.1, Pharsight Corporation, United States). The appropriate PK models were selected based on the MBF concentrations plotted on semi-logarithmic graphs. Moreover, the same models for PK analysis were selected after i.v and i.m administrations.

### PK-PD Integration Modeling Analysis

For PK-PD indices selection for an antibiotic, these three indices (*f*AUC/MIC, *f*C_max_/MIC and *f*T > MIC) are standardized (Nielsen and Friberg, 2013). AUC is the area under the concentration-time curve, C_max_ is the highest concentration reached (the peak), and T > MIC is the cumulative percentage of a 24 h period that the concentration is above MIC, *f* is the free and unbound fraction of the drug. The best PK-PD index for a certain drug-bacteria combination is determined by plotting the value of an efficacy endpoint (typically log10 CFU/ml after 24 h of treatment) versus the magnitude of each of the three PK-PD indices. It has been reported that AUC/MIC could be the right and optimal PK-PD index for MBF based on the previously published study (Sidhu et al., 2010; Jian et al., 2015; Lei et al., 2017a; Zeng et al., 2017). The best PK-PD index of AUC/MIC was selected to use in this study. The applicable model equation for this study was shown as (Equation 1):

$$\begin{array}{c} {E=E}_{\max}\cdot \left(1-\frac{C^{N}}{C^{N}+{\mathrm{EC}}_{50}^{N}}\right) \end{array}$$

E is the summary PD endpoint, C is one of the three PK-PD indices as defined above, E_max_ is the maximum effect in Lg change after 24h compared to the initial inoculums, EC_50_ is the magnitude of C that is needed to achieve 50% of E_max_, N is the sigmoidicity factor. The figure of illustration relationship between E and C is provided, and the Y-axis means the effect of the antimicrobial agent measured as the log10 difference in bacterial number before and after 24 h incubation, and the X-axis means one of the three PK-PD indices as defined above.

### Dose Estimations

To assume an optimal regime, the following formula was used:

$$\begin{array}{c} \mathrm{Dose}=\frac{(AUC/MIC)\cdot {\mathrm{MIC}}_{90}\cdot \mathrm{CL}}{fu\cdot F} \end{array}$$

Where AUC/MIC represented the targeted endpoint for optimal effect in hours, MIC_90_ represented the MIC value containing 90% isolates in the population of strains, CL represented clearance ration, *fu* represented the free drug in serum, and F represented the value of bioavailability.

The probabilities of distribution of predicted daily dosages were conducted for 100000 trails to achieve 50 and 90 % target attainment rates (TAR) for bacteriostatic, bactericidal and bacterial elimination effects by using Monte Carlo Simulations in Oracle Ball (Oracle Corporation, Redwood Shores, CA, United States).

### Statistical Analysis

Data were shown as mean ± SD and calculated from 6 to 8 independent experiments. The MIC_50_ and MIC_90_ were obtained by using SPSS software. The statistical analyses were done by the Student’s *t*-test and ANOVA using Prism software (Graphpad Software Inc., London, United Kingdom). *P*-values < 0.05 indicated statistically significant differences.

## Results

### MIC Distribution of MBF against *SS*

**FIGURE 1:**
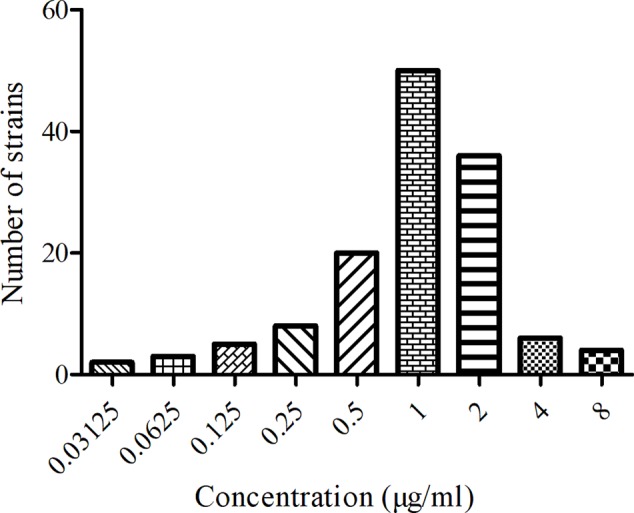
The MIC distribution of MBF against 134 clinical isolates *SS* from pigs.

The MIC distribution and values of MBF against the 134 *SS* strains were shown in **Figure 1**. The MIC values ranged from 0.03125 to 8 μg/ml. The MIC_50_ and MIC_90_ of these strains were calculated to be 1 and 2 μg/ml respectively, suggesting MBF displayed a potent antibacterial effect against *SS*. According to the MIC_90_ value and the result of serotype identification (not shown), we selected the HB2 serotype, with similar MIC and MIC_90_ values, to study the *in vitro* and *ex vivo* antimicrobial effect of MBF.

### MIC, MBC, and MPC Detection of MBF against HB2

The HB2 with the MIC close to the MIC_90_ was chosen as the representative isolate for PD study *in vitro* and *ex vivo*. The MIC and MBC of HB2 were determined to be 1 and 4 μg/ml in TSB, and 2 and 4 μg/ml in pig serum, respectively. The MBC/MIC ratios were 4 and 2 *in vitro* and *ex vivo*, respectively. Moreover, the MPC of MBF against HB2 was 2.56 μg/ml. These result revealed that MBF might have a relatively gentle concentration-effect against *SS*.

### Growth and Killing-Time Curves of MBF against HB2 *in Vitro* and *ex Vivo*

**FIGURE 2:**
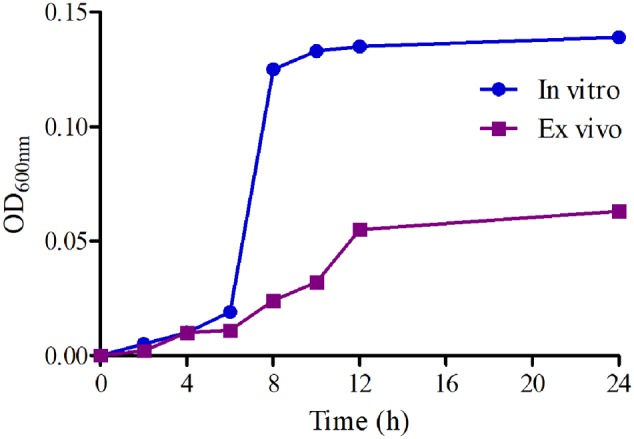
The growth-time curves of HB2 in TSB and serum.

The growth-time curves of HB2 in TSB and serum are illustrated in **Figure 2**. According to the growth-time curve profiles, the logarithmic phases of HB2 in TSB and serum were from 2–10 h and 2–12 h, respectively whose bacteria amount (OD values) were increased logarithmically. The total bacterial amount and growth rate were higher in TSB than in serum. The findings suggest that serum can inhibit bacterial growth.

**FIGURE 3:**
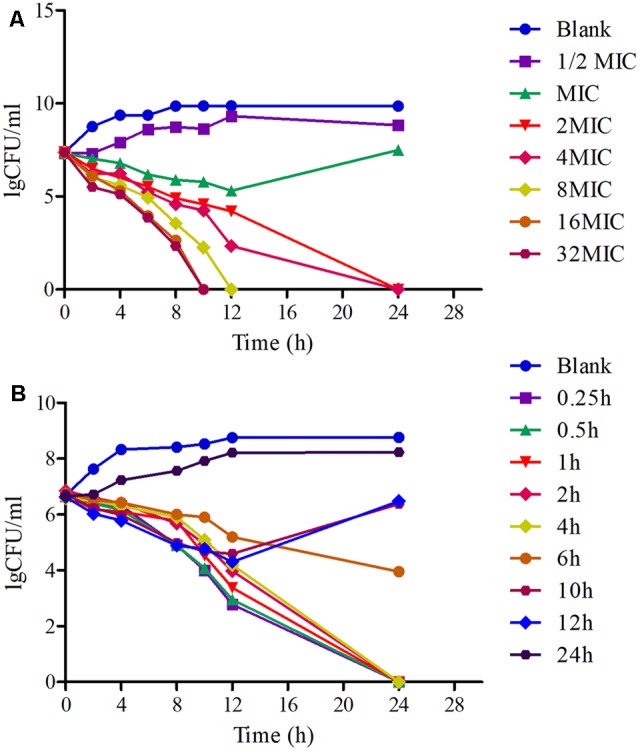
The killing-time curves of MBF against *SS* (HB2) *in vitro* (TSB) and *ex vivo* (Serum). **(A)** Representing the curve in TSB; **(B)** representing the curve in serum.

The killing-time curves of MBF against HB2 *in vitro* and *ex vivo* are illustrated in **Figures 3A,B**. According to the profiles of curves *in vitro* and *ex vivo*, MBF displays a concentration-dependent bactericidal activity against *SS* as increasing drug concentrations induced more rapid and radical bactericidal effect. Moreover, both the *in vitro* and *ex vivo* curves show the same bactericidal tendency; after exposure to 1 MIC or less of MBF, the bacteria could recover growth after 12 h. However, after exposure to a greater concentration than 1 MIC of MBF for 24 h, the bacterial CFU values were markedly reduced (<30 CFU) (**Figure 3A**). The bacterial CFU values were also markedly reduced (<30 CFU) in the serum from pigs of the PK experiment whose MBF concentration was above 2 MIC (**Figure 3B**). The killing-time curves *in vitro* and *ex vivo* were analogical. These findings suggest that MBF has a concentration-dependent action against *SS* both *in vitro* and *ex vivo*.

### PK Analysis of MBF in Serum after Oral and i.v Administration

**FIGURE 4:**
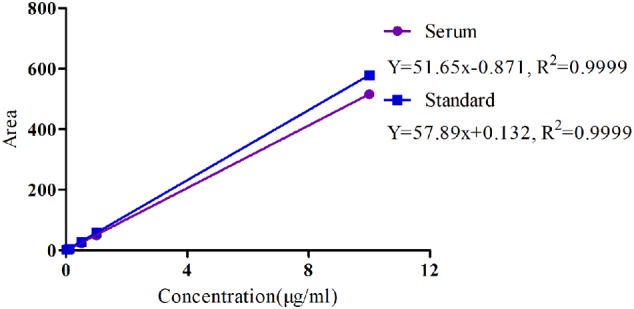
The standard curve of MBF in serum and standard liquid ranged from 0.02 to 10 μg/ml.

The specificity of MBF detection methods in plasma was good and suitable and there was no any interference observed in the chromatograms. Moreover, the linear range of the standard curve of MBF ranged from 0.02 to 10 μg/ml were shown in **Figure 4**. The coefficient of determination (*R*^2^) of standard curves from 0.05 to 10 μg/ml was 0.9999, and the inter-day and intra-day coefficient variation were <10% (**Figure 4**). Moreover, the recovery ratios values were in the range of 90 ± 2.23 to 94 ± 3.12 % in serum for MBF detection. The lower limit of detectability (LLOD) and lower limit of quantitation (LLOQ) were 0.02 and 0.05 μg/ml in plasma (**Figure 4**). In addition, the typical regression equation was *y* = 51.65 x – 0.087, *R*^2^ = 0.9999 in serum, and *y* = 57.89 x + 0.132, *R*^2^ = 0.9999 in standard (**Figure 4**).

**FIGURE 5:**
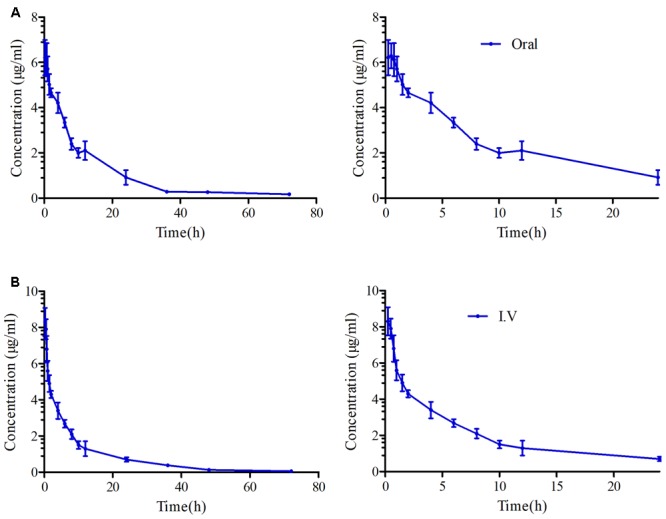
The curves of MBF concentration-time in serum after oral and i.v administration at a dose of 8 mg/kg. **(A)** Representing the curve in serum after oral administration at 24 and 72 h, **(B)** representing the curve in serum after i.v administration at 24 and 72 h.

For the pooled serum samples of the 8 pigs of the PK experiment, the percentage free drug concentration was 92 ± 4.3%. The mean MBF concentration-time profiles were exhibited in **Figure 5** following oral and i.v administrations of MBF. In addition, the principal PK parameters were exhibited in **Table 1** with using non-compartment models analysis after i.v and i.m administrations. The C_max_, AUC_0-24 h_, AUC_0-∞_, Ke, t_1/2ke_, T_max_, Cl_b_ and MRT values were 6.28 μg/ml, 60.30, 88.94 μg.h/ml, 0.056 h^-1^, 12.48 h, 0.75 h, 0.104 L/h and 16.27 h after oral administration, and 66.55, 94.40 μg.h/ml, 0.046 h^-1^, 15.16 h, 0.085 L/h and18.93 h after i.v administration, respectively (**Table 1**). Furthermore, the bioavailability of MBF was calculated to be 94.21% after a single 8 mg/kg oral administration.

**Table 1:** The PK parameters after oral and i.v administrations (8 mg/kg) in pigs (mean ± SD).

Parameters	Unites	Oral	i.v
Ke	h^-1^	0.056 ± 0.015	0.046 ± 0.011
*t*_1/2ke_	h	12.48 ± 2.15	15.16 ± 2.43
Cl_b_	L/h	0.104 ± 0.02	0.085 ± 0.03
AUC_0-∞_	μg^⋅^h/ml	88.94 ± 6.47	94.40 ± 11.38
AUC_0-24 h_	μg^⋅^h/ml	60.30 ± 6.47	66.55 ± 7.12
C_max_	μg /ml	6.28 ± 0.92	–
T_max_	h	0.75 ± 0.13	–
MRT	h	16.27 ± 2.47	18.93 ± 3.37
F	–	94.21%	–

*Ke, presented elimination rate constant; *t*_*1/2ke*_, presented elimination half-life; Cl_*b*_, presented body clearance; AUC_*0*-∞_, presented area under the curve from 0 to ∞; AUC_*0*-*24 h*_, presented area under the curve from 0 to 24 h; C_*max*_, peak concentration; T_*max*_, the time to reach peak concentration; MRT, presented mean residence time; F, the bioavailability.*

### PK-PD Integration Modeling

The PK-PD parameters of AUC_0-24h_/MIC, AUC_0-24h_/MPC, C_max_/MIC and C_max_/MPC received from PK and PD data in serum were exhibited in **Table 2**. Moreover, the estimated AUC_0-24h_/MIC, AUC_0-24h_/MPC, C_max_/MIC, and C_max_/MPC values of MBF against *SS* were 30.06, 23.47 h, 3.14 and 2.45, respectively (**Table 2**). Moreover, the time values for the MBF higher than the *ex vivo* MIC (T > MIC) and MPC (T > MPC) in serum were 11.02 and 7.56 h (**Table 2**). These results revealed that MBF could have high concentrations (above three times to MIC) and long antibacterial effect (over 24 h), and also reach the high concentration above MPC for 7 h. These indicated MBF had a strong antibacterial manner against *SS in vitro* and *ex vivo*.

**Table 2:** The PK-PD parameters for MBF against HB2 in serum.

Parameters	Unites	Mean ±*SD*
C_max_/MIC	–	3.14 ± 0.76
T > MIC	H	11.02 ± 1.43
C_max_/MPC	–	2.45 ± 0.34
T > MPC	h	7.56 ± 0.96
AUC_0-24 h_/MIC	h	30.06 ± 4.35
AUC_0-24 h_/MPC	h	23.47 ± 2.64

**FIGURE 6:**
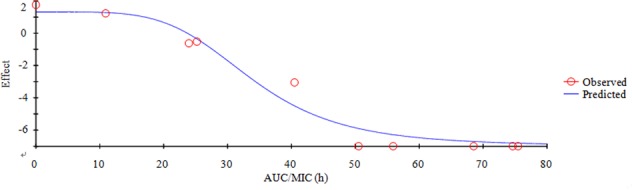
Plots of AUC/MIC ratios in *ex vivo* versus the lg decreased the amount of HB2 within 24 h.

Based on the previously described reports for quinolones and the profiles of the killing-time curve of MBF against *SS*, it could be obviously to estimate MBF had a concentration-dependent action. Thus, the AUC_0-24 h_/MIC or C_max_/MIC could be selected as PK-PD index. PK-PD index approach had become the gold standard for evaluation PK-PD of antibiotics and to guide the establishment of dosing regimens. These three indexes were highly correlated, making it important to have data from several different dosing regimens to be able to distinguish between them. According to the previously published study, it has been demonstrated that AUC/MIC could be regarded as the right and optimal PK-PD index for MBF (Sidhu et al., 2010; Jian et al., 2015; Lei et al., 2017a; Zeng et al., 2017). Therefore, the best PK-PD index of AUC/MIC was selected to use for proposing the optimal dose in this study. The antibacterial activity of MBF against HB2 was tested in the serum of those pigs used from the PK experiment (at 0.25–24 h after oral administration of 8 mg/kg MBF). Based on the inhibitory sigmoid E_max_ model, the relationship between antimicrobial efficacy and the *ex vivo* AUC_0-24 h_/MIC ratios were simulated with an optimal *R*^2^ (0.989) compared with others parameters, and the calculated model parameters are shown in **Figure 6** and **Table 3**. The AUC_0-24 h_/MIC ratios were calculated for bacteriostatic effect (*E* = 0, 25.23 h), bactericidal effect (*E* = -3, 35.64 h), and bacterial elimination effect (*E* = -4, 39.71 h) (**Table 3**).

**Table 3:** PK-PD modeling of MBF *ex vivo*.

Parameters	Unites	Mean ±*SD*
E_max_	LgCFU/ml	1.67 ± 0.34
EC_50_	h	33.82 ± 2.56
N	–	4.70 ± 0.82
AUC_0-24 h_/MIC for bacteriostatic (E = 0)	h	25.23 ± 4.25
AUC_0-24 h_/MIC for bactericidal (E = -3)	h	35.64 ± 4.85
AUC_0-24 h_/MIC for eradication (E = -4)	h	39.71 ± 6.45

*E_*max*_, represented the maximum effect in Lg change after 24 h compared to the initial inoculums; EC_*50*_, presented the value to achieve 50% maximal antibacterial effect; N, presented the Hill coefficient; Hill, the value of curve slop.*

### The Predicted Doses Evaluation

**FIGURE 7:**
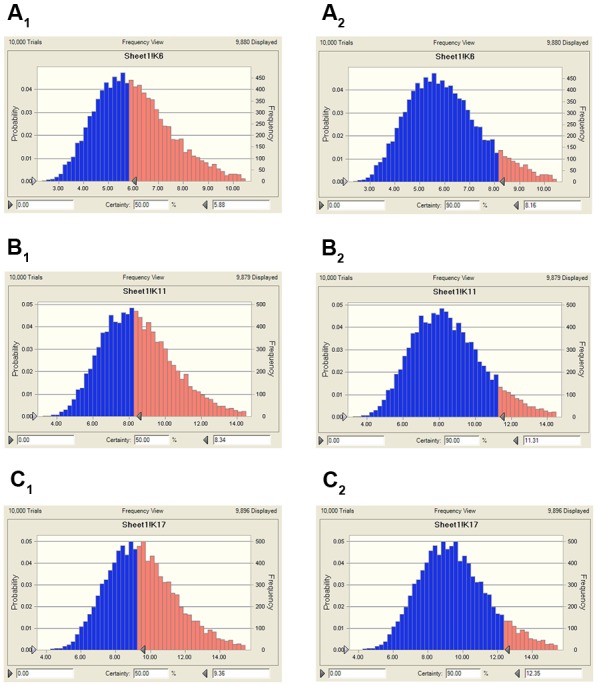
The predicted population dosage of MBF against SS (HB2) for 50% and 90% TAR. **(A_1_)**, representing the predicted dosage for bacteriostatic activity at 50% TAR, **(A_2_)**, representing the predicted dosage for bacteriostatic activity at 90% TAR, **(B_1_)**, representing the predicted dosage for bactericidal activity at 50% TAR, **(B_2_)**, representing the predicted dosage for bactericidal activity at 90% TAR, **(C_1_)**, representing the predicted dosage for elimination activity at 50% TAR, **(C_2_)**, representing the predicted dosage for elimination activity at 90% TAR.

Using the data generated here, we estimated the optimal once-daily doses of MBF (**Table 4**). The distribution of predicted daily population dosage (AUC_0-24h_/MIC) values of MBF curing *SS* for 50 and 90% target were calculated and observed in **Table 4** and **Figure 7**. Based on dose equations and Monte Carlo Simulations, we predicted the MBF dosages needed for bacteriostatic, bactericidal, and elimination activity curing *SS* HB2 as 5.88, 8.34 and 9.36 mg/kg.bw for 50% target, and 8.16, 11.31 and 12.35 mg/kg.bw for 90% target, respectively in **Table 4**.

**Table 4:** The predicted daily dosages for MBF curing *SS.*

Predicted doses (mg/kg.bw)	Target ratios	
	50%	90%
Bacteriostatic (*E* = 0)	5.88	8.16
Bactericidal (*E* = -3)	8.34	11.31
Eradication (*E* = -4)	9.36	12.35

## Discussion

The abuse of antibiotics leads to bacterial resistance. For the classical MBF fluoroquinolone, misuse led to an increase in MIC_90_ versus *SS* (0.5–1 μg/ml) and multiple MBF resistant isolates (El Garch et al., 2017; Meunier et al., 2004; Kroemer et al., 2012; Kroemer et al., 2014). Here, we estimated the MIC_50_ and MIC_90_ of MBF against 134 strains of *SS*, which ranged between 0.5 and 1 μg/ml and 6.8% of these strains were MBF-resistant (**Figure 1**). These values are similar to previous reports, which have identified fluoroquinolone-resistant *SS* strains from multiple countries (Escudero et al., 2007; Escudero et al., 2011; Yao et al., 2014). Such resistance mechanisms are complex and cross-resistance is common. Therefore, it is crucial to use antibiotics reasonably and to determine optimal doses.

Here, we show that MBF has good *in vitro* antibacterial activity against *SS* (MIC_90_ = 2 μg/ml). HB2, a highly pathogenic clinical isolate serotype 2, was selected for PD analyses. In previously published reports, the pathogen was selected from 6 to 10 isolates of target animals or simply a standard strain (Yohannes et al., 2015; Hossain et al., 2017; Zhou et al., 2017). HB2 is considered to be an emerging and virulent human pathogen that is becoming increasingly potent and is particularly prevalent in Southeast Asian countries (Yu et al., 2006; Feng et al., 2010, 2014; Gottschalk et al., 2010b). Moreover, the MIC value of HB2 was equal to the MIC_90_ of these isolates from pigs, and it also had been verified the virulent in mice. Thus, compared to previous studies, the HB2 strain used in the study could be a more favorable representation for PD. Here, the total bacteria counts for HB2 were lower in serum than in TSB, whereas the MIC values were similar (**Figure 2**). This might be due to serum effect potency, as was reported by Toutain (Toutain et al., 2017). We show that the antibacterial activity of MBF against HB2 is concentration-dependent (**Figure 3**). We found that MBF concentrations greater than 2 MIC could eliminate SS within 24 h, both *in vitro* and *ex vivo* (serum) (**Figure 3**). These findings are similar to those previously reported for MBF against *E.coli, Pasteurella multocida*, and *Haemophilus parasuis* (Cao et al., 2015; Sun et al., 2015; Lei et al., 2017a). Taken together, our data show that MBF has strong antibacterial effect against *SS*.

PK-PD integration modeling can be used to select rational dose regimes in veterinary medicine (Toutain et al., 2002), and the drug concentration detection at the infection site is preferred for PK-PD modeling (Lei et al., 2017a). However, for respiratory tract infection, especially for extracellular bacteria (e.g., *SS, Haemophilus parasuis* and *Pasteurella multocida*), the target tissue (PELF) may be suitable for PK-PD because of its high drug concentrations. However, PELF is unable to maintain effective local extracellular concentration and the measuring PELF concentration could be very difficult and inconvenient. (Nowakowski et al., 2004; Villarino et al., 2013a,b; Toutain et al., 2016). In addition, it has been reported that MBF has a high bioavailability (nearly 100%) after oral or intramuscular injection (Ding et al., 2010). Here, we estimated MBF bioavailability as 97.74% after an oral dose of 8 mg/kg. Thus, the serum could be regarded as an optimal site for PK and PD studies of MBF. PK-PD modeling studies have been done for MBF against *Eshcerichia coli, Haemophilus parasuis*, and *Pasteurella multocida* in calve, sheep, broiler chicken, turtles, beagle, and pigs (Sidhu et al., 2010; Vallé et al., 2012; Potter et al., 2013; Qu et al., 2015; Sun et al., 2015; Yohannes et al., 2015; Lei et al., 2017a). However, to our knowledge, prior to our study, no PK-PD integration modeling analyses have been done for MBF against *SS*.

The daily doses (2 mg/kg) of MBF for 3–5 days were clinically effective for the treatment of respiratory diseases, and that single doses of 8 and 10 mg/kg in pigs and cattle, respectively, were also effective (Vallé et al., 2012; Schneider et al., 2014; Grandemange et al., 2017). Here we used an oral and single dose of MBF (8 mg/kg) against *SS*. The AUC_0-∞_ value after an oral dose of 8 mg/kg (88.94 μg.h/ml) was found much higher than that after an oral dose of 2 mg/kg (14.67 μg.h/ml) (Lei et al., 2017a), whereas the *t*_1/2ke_ value (12.48 h) was similar that of previous reports in pigs (23.14 and 30.67 h) (Ding et al., 2010; Lei et al., 2017a). Moreover, the *t*_1/2ke_ value (12.48 h), C_max_ (6.28 μg/ml), T_max_ (0.75 h), AUC_0-∞_ (88.94 μg.h/ml) and F (94.21%) were all similar to those C_max_ (5.86 μg/ml), T_max_ (0.93 h), AUC_0-∞_(79.9 μg.h/ml), F (89.6%) in the published report by M. Schneider at the same dose administration of 8 mg/kg (Schneider et al., 2014). The clearance values of 0.12 L/h reported by Ding and 0.092 L/h reported by M. Schneider were similar to the Cl_b_ of 0.104 observed after oral administration in this study but higher than that (0.085 L/h) after i.v administration. The difference might be explained by the administration methods and there was no absorption phase in i.v administration. These were also the interpretation for the difference in ke, t_1/2_ and MRT parameters.

In general, fluoroquinolones are regarded to represent a concentration-dependent action, and the AUC_0-24 h_/MIC and C_max_/MIC ratios appear to be the appropriate parameters to predict their antimicrobial effect and to make comparisons with quinolones (Lode et al., 1998; Wise and Honeybourne, 1999).

An AUC_0-24h_/MIC ratio of >125 h and C_max_/MIC ratio of >10 are generally considered the best activity indicators for antibacterial agents with concentration-dependent killings, and these are also used as a threshold for a successful therapeutic outcome (Meinen et al., 1995; Anadón et al., 1999; Toutain et al., 2002). However, these thresholds might be different for different fluoroquinolones, and the distinct AUC_0-24 h_/MIC or C_max_/MIC ratios were required for clinical cure depending on the detailed host or pathogen (Heinen, 2002). An AUC_0-24 h_/MIC ratio of 46 h has been reported for the bactericidal activity of MBF against *Mannheimia haemolytica*, as well as of 48 h for MBF against *Haemophilus parasuis*, and 24 h for MBF against *Escherichia coli* (Aliabadi and Lees, 2002; Sun et al., 2015; Lei et al., 2017a). Therefore, it was very important to select the PK-PD indices of fluoroquinolones. Here we report C_max_/MIC, C_max_/MPC, AUC_0-24 h_/MIC, and AUC_0-24h_/MPC values of 3.14, 2.45, 30.06, and 23.47 h, respectively (**Table 2**). An appropriate E_max_ model was selected for PK-PD integration modeling and dosage prediction, and it represented a suitable correlation (0.989) between the predicted and observed *ex vivo* antibacterial effect of MBF against *SS* (**Figure 6**). The Sigmoid E_max_ in the Equation1 was the best applicable with a highest favorable correlation (0.989) than others modeling for PD study which was different from the previously published report of MBF against *E. coli* (Lei et al., 2017a). The *ex vivo* AUC_0-24h_/MIC ratios of MBF against *SS* serotype 2 (HB2) for bactericidal and eradication effect were 35.64 and 39.71 h respectively (**Table 3**), which were higher than the *ex vivo* AUC_0-24 h_/MIC (30.06 h) after oral administration of a single dose of 8 mg/kg (**Table 2**). These results suggested that the recommended 8 mg/kg dose might not guarantee clinical efficacy against infections associated with the *SS* serotype 2. According to the PK-PD data *in vitro* and *ex vivo*, the Monte Carlo simulation could predict dosage for clinical use with 50 and 90% TAR (Nielsen and Friberg, 2013; Dorey et al., 2017). Based on the dose estimation equation and Monte Carlo simulations, the predicted daily doses of MBF against *SS* serotype 2 for bacteriostatic, bactericidal, and eradication activity were 5.88, 8.34, and 9.36 mg/kg for 50% targets, and 8.16, 11.31, and 12.35 mg/kg for 90% targets, respectively (**Figure 7** and **Table 4**). However, it should be paid an attention that the bacterial endpoint *in vivo* might differ from the predicted doses calculated from *ex vivo* data since the animals’ immune system may play an important role in bacterial eradication (Garrison et al., 2002; Nielsen and Friberg, 2013; Lei et al., 2017a; Yan et al., 2017). In addition, the calculated PK and PD data from small sample size could not support the conclusions in this study. Therefore, these predicted daily dosages should also be verified in clinical practice.

## Conclusion

It has been demonstrated that the misuse of antibiotics increases antimicrobial resistance (Nguyen et al., 2012; Zhang et al., 2013). Our data demonstrate that a single oral dosage of MBF (8 mg/kg) is insufficient to have bactericidal effect against *SS*; a slightly higher dose of 11.31 mg/kg would be required, whereas 12.35 mg/kg would be required for eradication. According to the EMA, the desmethyl and N-oxide metabolites of MBF were considered to have anti-microbiological activity equal to that of MBF, and the conclusion may be also appropriate for these metabolites of MBF. These dose estimates should now be validated in clinical practice.

## Author Contributions

QL and JC conceived this work. ZL and JC designed the experiment. BY and ZL performed the experiments. ZL wrote the manuscript. QH, HK, and JC improved the language. All authors reviewed the manuscript.

## Conflict of Interest Statement

The authors declare that the research was conducted in the absence of any commercial or financial relationships that could be construed as a potential conflict of interest.
